# Differences in Inhibitory Control and Resting Brain Metabolism between Older Chronic Users of Tetrahydrocannabinol (THC) or Cannabidiol (CBD)—A Pilot Study

**DOI:** 10.3390/brainsci12070819

**Published:** 2022-06-23

**Authors:** Thorsten Rudroff, Craig D. Workman, Phillip E. Gander, Justin R. Deters, Laura L. Boles Ponto

**Affiliations:** 1 Department of Health and Human Physiology, University of Iowa, Iowa City, IA 52242, USA; craig-workman@uiowa.edu (C.D.W.); justin-deters@uiowa.edu (J.R.D.); 2 Department of Neurology, University of Iowa, Iowa City, IA 52242, USA; 3 Department of Radiology, University of Iowa, Iowa City, IA 52242, USA; phillip-gander@uiowa.edu (P.E.G.); laura-ponto@uiowa.edu (L.L.B.P.)

**Keywords:** THC, CBD, positron emission tomography, Flanker Task, aging

## Abstract

Δ9-Tetrahydrocannabinol is the main psychoactive component of cannabis and cannabidiol is purportedly responsible for many of the medicinal benefits. The effects of Δ9-tetrahydrocannabinol and cannabidiol in younger populations have been well studied; however, motor function, cognitive function, and cerebral glucose metabolism in older adults have not been extensively researched. The purpose of this study was to assess differences in cognitive function, motor function, and cerebral glucose metabolism (assessed via [^18^F]-fluorodeoxyglucose positron emission tomography) in older adults chronically using Δ9-tetrahydrocannabinol, cannabidiol, and non-using controls. Eight Δ9-tetrahydrocannabinol users (59.3 ± 5.7 years), five cannabidiol users (54.6 ± 2.1 years), and 16 non-users (58.2 ± 16.9 years) participated. Subjects underwent resting scans and performed cognitive testing (reaction time, Flanker Inhibitory Control and Attention Test), motor testing (hand/arm function, gait), and balance testing. Δ9-tetrahydrocannabinol users performed worse than both cannabidiol users and non-users on the Flanker Test but were similar on all other cognitive and motor tasks. Δ9-tetrahydrocannabinol users also had lower global metabolism and relative hypermetabolism in the bilateral amygdala, cerebellum, and brainstem. Chronic use of Δ9-tetrahydrocannabinol in older adults might negatively influence inhibitory control and alter brain activity. Future longitudinal studies with larger sample sizes investigating multiple Δ9-tetrahydrocannabinol:cannabidiol ratios on functional outcomes and cerebral glucose metabolism in older adults are necessary.

## 1. Introduction

Physiological changes predispose older adults more to geriatric conditions and chronic diseases, including falls and cognitive impairment. For example, inadvertent falls are commonplace in older adults and are a primary cause of morbidity, mortality, and decline in functional and disability status [1]. Moreover, cognitive impairment and falls are a “well-known couple”; older adults with moderate to severe cognitive impairment have twice the risk of falling (~60–80%) compared to cognitively normal older adults [2]. Importantly, the incidence of cannabis use for medicinal purposes has increased significantly among US adults aged 50 years and older [3,4]. Furthermore, the chances of developing a disability or disease responsive to medical cannabis, e.g., nausea, cachexia, pain, and cancer-related conditions [5] increases with age, further increasing the prospect of use. With expanded accessibility and use of medical cannabis by older adults, a rigorous evaluation of the benefits and risks in this population is required.

The *Cannabis sativa* plant is comprised of more than 100 compounds, of which Δ9-tetrahydrocannabinol (THC) and cannabidiol (CBD) are the most prevalent. THC is the primary psychoactive component and has historically been associated with public health risks and small-moderate therapeutic efficacy [6]. In addition, THC might also provoke acute psychosis and negatively influence executive function [6,7]. On the contrary, some research has indicated anti-epileptic [8], anti-spastic [9], and analgesic [10] properties of THC. CBD is non-intoxicating [11] and might be anxiolytic, anti-epileptic, anti-inflammatory, and neuroprotective [12]. CBD could also diminish the negative side effects of THC [13]. Although CBD has an encouraging safety profile [14], it still has potential for adverse effects like liver toxicity [15] and drug interactions [16]. Both THC and CBD act via the modulation of cannabinoid receptors CB1 (primarily expressed in the central nervous system) and CB2 (primarily expressed in the peripheral nervous system) and their efficacy/bioavailability are dependent on route of administration (e.g., inhalation vs. ingestion) [6,11].

Cannabis use, particularly THC-dominant products, has been shown to decrease cognitive function, with effects that might stay later in life [17]. Therefore, the physiological consequences of chronic cannabis use might further increase cognitive impairment and associated falls in older adults [18,19]. Additionally, cannabis may have an effect on cognitive-motor skills and functional brain mechanisms that alter coordinated movement in habitual users. For instance, one study [20] described reduced activity in the supplementary motor area in regular cannabis users while they performed a motor function test. Thus, disturbances of these networks, including reduced neural activity in the frontal lobe, might cause fall risks via impaired motor control. Furthermore, Workman, et al. [18] reported slower gait speed, poorer unipedal balance, and higher fall risk in older chronic medical cannabis users vs. non-users. However, it remains unclear whether these impairments might be attributed to the THC or CBD content of the cannabis consumed by the users.

Knowledge about the role of chronic use of THC and CBD in older adults on functional performance and brain health is limited because previous work has primarily studied young and middle-aged cohorts, with only a few small observational studies in older adults. Comparing older cannabis users to younger users is challenging because older adults take up cannabis constituents differently than younger people and may experience the effects in a different way [21,22]. Possible health risks are related with cannabis use due to physiological aging, concomitant use of prescribed medications, and increased number of comorbid conditions. Furthermore, older adults may feel increased vulnerability to adverse drug reactions because of reduced hepatic drug clearance and renal elimination [23]. Adult cohorts under the age of 65 have demonstrated that high THC use resulted in acute impairments of learning, attention, and memory [24,25,26]. However, it is unclear whether these cognitive impairments persist after acute consumption has shifted to chronic use (i.e., using ≥ 6 months).

Furthermore, the impact of chronic medical cannabis use on cognitive function varies with the age of cannabis-use onset, duration of use, frequency of use, and duration of abstinence [26,27]. In addition, a variety of neurodegenerative processes occur with age, including decreases in whole brain, hippocampal, and temporal volumes [28]. Moreover, older adults experience decreases in endocannabinoid system function, including decreased cannabinoid receptor 1 (CB1) binding and reduced concentrations of some endocannabinoids [29]. Cannabis might also exacerbate the decrease in visual skills and cognitive-processing speed associated with normal aging, which could have consequences for raising the risk of falls or other injuries [30,31]. However, this might be specific to THC-dominant cannabis, as a recent review by Batalla, et al. [32] stated that CBD has opposite effects to THC in cognitive outcomes after acute administration. Thus, results from young adult studies might be informative for driving initial older adult investigations and hypotheses, but the functional and structural differences in the brains of older adults will likely result in varying cognitive sequalae of cannabis use compared to younger adults.

Positron emission tomography (PET) with [^18^F]-Fluorodeoxyglucose (FDG) can be used to assess brain activity and may provide insight to the effects of THC and CBD [33,34]. Importantly, previous FDG-PET studies have linked lower cerebral glucose uptake to increased fall risk [35] and mild cognitive impairment in patients with Alzheimer disease [36] and multiple sclerosis [37]. It is known that cerebral blood flow and cerebral glucose metabolism are correlated [38,39,40]. A review by Bloomfield, et al. [7] indicated that chronic THC use was related with decreased blood flow and hypometabolism in several brain regions and acute CBD administration was correlated with decreased blood flow in limbic and paralimbic areas. However, no studies have investigated brain metabolism or blood flow changes after chronic CBD use.

Although cannabis has achieved a lot of attention, the chronic effects of THC and CBD use in older adults, and the effect of these substrates on cognition, motor function, and cerebral glucose metabolism are largely unknown. The purpose of this exploratory, cross-sectional, observational pilot study was to elucidate differences in motor function, cognitive function, and cerebral glucose metabolism in older non-using adults and those chronically using THC- and CBD-dominant cannabis for medical reasons. The hypothesis was that older adults who chronically use THC-dominant products would perform worse than both CBD-dominant users and non-users on cognitive and motor tasks. Moreover, it was hypothesized that these findings would be accompanied by diverse cerebral glucose metabolism patterns between the groups.

## 2. Materials and Methods

### 2.1. Subjects

Eight THC-dominant and five CBD-dominant medical cannabis users (THC-users and CBD-users, respectively), and sixteen age- and sex-matched controls (non-users; NU) were recruited (see Table 1 for subject demographics). Inclusion criteria were (1) between 50–80 years old, (2) in the Iowa Medical Cannabidiol program and have been using cannabis products for at least 6 months (users) or have not used cannabis in ≥ 10 years (NU), (3) able to do the protocol based on past medical history, (4) able to understand the protocol (responded to questions about the study after reading the consent form), (5) able to use and be contacted by telephone, (6) could to read, speak, understand, and complete questionnaires in English, and (7) willing to abstain from cannabis use for at least 4 h prior to testing (THC and CBD users). Exclusion criteria included: (1) pregnancy, (2) history of traumatic brain injury, (3) presence of pacemakers, aneurysm clips, artificial heart valves, or metallic prostheses. This study was approved by the Institutional Review Board at the University of Iowa and was performed in accordance with the Declaration of Helsinki. All subjects provided written consent prior to participation.

### 2.2. Experimental Protocol

Subjects accomplished two experimental sessions. Session 1 began with a urine test (iScreen IS1 THC dipstick; Alere Toxicology, Portsmouth, VA, USA) to identify the presence of cannabis and ensure accurate group assignment (user or NU). Product labels containing THC and CBD content of the using groups also verified cannabis group assignment. Subjects then completed the 9-Hole Peg Test (9HPT), the Flanker Inhibitory Control and Attention Test, the Deary-Liewald reaction time (RT) tasks (simple and choice RT), a 30 m walk test, Item 14 of the Berg Balance Scale (BBS), question 1 of the Activities Balance Confidence (ABC-1) scale, maximal handgrip strength testing, and static posturography. Session 2 consisted of structural magnetic resonance imaging (MRI) and an FDG-PET scan. Subjects fasted for a minimum of 6 h before Session 2 to ensure blood glucose was ≤200 mg/dL for FDG administration and PET imaging [41,42]. Subjects in the THC and CBD groups also confirmed that their previous cannabis consumption was >4 h prior to testing for each session.

### 2.3. Measurements

#### 2.3.1. Arm and Hand Function

The 9HPT assesses upper extremity function [43]. The test board consists of nine holes and a small box containing nine pegs. Each peg was placed, one at a time, into an empty hole as fast as possible. After each peg was inserted, the subjects took the pegs, one at a time, and dropped them into the box. The time required to place and remove all nine pegs was the outcome measure. Timing started when the subjects touched the first peg and ended when they dropped the last peg in the box. Both the hands were tested two times in an interchanging order. Participants began with the dominant hand. The time to carry out each trial was recorded and averaged for each hand.

#### 2.3.2. Cognitive Function

Cognitive function was calculated using the Deary-Liewald simple and choice RT task [44] and the Flanker Inhibitory Control and Attention Test [45]. During the simple RT task, subjects pressed the space bar on a laptop computer keyboard (Dell Latitude 7490, Dell, Round Rock, TX, USA) as quickly as possible when a black “X” appeared in a white box located in the center of the computer screen. Eight familiarization trials were performed before 20 measurement trials. The time between the X appearing on the screen and the subjects pressing the space bar was recorded and averaged over the 20 trials. During the choice RT tasks, four white boxes were positioned horizontally on the computer monitor and corresponded to a specific key on the laptop keyboard. The “Z” key corresponded to the far-left box, the “X” key to the box second from the left, the “comma” (,) key to the box second from the right, and the “full stop” (.) key to the box on the far right. The subjects positioned four of their fingers (i.e., two from each hand) above, but not touching, each key before the task began and were required to act as rapidly and accurately as possible to a black “X” that randomly appeared in one of the four boxes by pressing the corresponding key. Eight familiarization trials were performed before 40 measurement trials. The accuracy of each trial and the time between the cross appearing on the screen and the subjects pressing the corresponding key was recorded and averaged over the 40 trials. The inter-stimulus interval for the cross appearance ranged randomly between 1 and 3 s for both the simple and choice RT tasks.

The Flanker Inhibitory Control and Attention Test evaluates the capacity to inhibit visual attention to extraneous task elements [45]. During this task, a central arrow was flanked on either side by two analogous arrows (five arrows total). The task requires subjects to signify the direction that the central arrow is pointing by pressing the “A” key for left and “L” key for right. A guide was placed over the laptop keyboard that left only the “A” and “L” keys visible to the subjects to account for any unfamiliarity the subjects may have had with a QWERTY keyboard; this guide also had a centrally located mark that served as the start or “home” position for each trial. Subjects were asked to start at the home mark, press the appropriate key in response to the displayed arrows, and then return their hand to the home mark as quickly and as accurately as possible. The subjects performed this task with their dominant hand only. During congruent trials, all five arrows faced the same direction (e.g., →→→→→), while the flanking arrows (i.e., those around the central, target arrow) faced in the opposite direction of the central arrow on incongruent trials (e.g., →→←→→). The time between the arrows appearing on the screen and the subjects pressing the corresponding key was recorded, and the difference between the RT on the congruent (FT-C) and incongruent (FT-I) trials (i.e., the Flanker Effect (FT-E)) was calculated.

#### 2.3.3. Gait

A 30 m walk test (30MWT) evaluated gait performance. Throughout this experiment, subjects were ordered to walk 30 m at their regular walking speed. The subjects wore OPAL sensors (APDM Wearable Technologies Inc., Portland, OR, USA) on their bilateral feet and wrists, their sternum, and lower back (i.e., ~L5) to objectively quantify temporospatial gait characteristics. A stopwatch and step counting were also used to measure stride and gait length variables, which were confirmed by two testers. The whole time to carry out the walk was documented as the primary outcome variable. Because the subjects were using cannabis for medical purposes (e.g., joint pain control), it was expected that gait performance might suffer with multiple trials. Thus, only a single 30MWT trial was performed.

#### 2.3.4. Fall Risk 

Fall risk was calculated using the Lajoie and Gallagher [46] model and includes scores on BBS-14 [47], ABC-1 [48], and simple RT [44], which were correlated with fall risk with 91% sensitivity and 97% specificity. For BBS-14, subjects were asked to stand on a single leg of their choice for at least 10 s [47], while ABC-1 assesses how confident the subjects were that they would not fall when going around their home on a scale from 0–100% [48]. The prediction of fall risk was calculated using the following formula [35]:

$$\frac{\exp\left(-7.519+0.026\;\left(\mathrm{simple}\;\mathrm{RT}\right)-0.071\;\left(\mathrm{ABC}-1\right)-2.139\;\left(\mathrm{BBS}-14\right)\right)}{1+\exp\left(-7.519+0.026\;\left(\mathrm{simple}\;\mathrm{RT}\right)-0.071\;\left(\mathrm{ABC}-1\right)-2.139\;\left(\mathrm{BBS}-14\right)\right)}\times 100$$

#### 2.3.5. Static Posturography

Static posturography was accomplished on a balance board (Balance Tracking Systems, San Diego, CA, USA). Subjects stood as still as possible on the platform for 60 s with their arms folded and eyes looking at a symbol on the wall. The outcome variables were the center of pressure (COP) path length in the anterior-posterior (AP) and medial-lateral (ML) directions and the area of an ellipse that encapsulated 95% of the 2D area explored (COP area).

#### 2.3.6. Handgrip Strength Testing 

Maximal voluntary handgrip strength was assessed with a hydraulic JAMAR 5030J1 hand dynamometer (Sammons Preston Rolyan, Bolingbrook, IL, USA). The dynamometer was customized for each subject to control for hand size and measurements were performed while the subjects were seated with their arm flexed to 90°. Both the non-dominant and dominant hands were examined three times in an alternating order. The participants began with the dominant hand. At least 30 s of rest was given between trials of same hand. The force produced from all three trials was recorded and averaged for each hand.

### 2.4. MRI/PET Scans 

An MRI was conducted on a GE 750W 3T scanner using a 48-channel head coil. Anatomical images included volumetric sagittal T1 MP-RAGE (TI = 900 ms, TE = 8.5 ms, flip angle = 8°, FOV = 256 × 256 × 192 mm, matrix = 256 × 256 × 192, bandwidth = 250 Hz/pixel, acceleration = 2) and sagittal T2 CUBE (TE = 60 ms, TR = 2500 ms, echo train length = 140, FOV = 256 × 256 × 192 mm, matrix = 256 × 256 × 192, bandwidth = 500 Hz/pixel, acceleration = 2) scans acquired using prospective motion correction (PROMO) and a 1.0 mm isotropic spatial resolution.

Prior to PET imaging, 5 mCi ± 10% FDG was administered via intravenous injection and tracer uptake occurred in a quiet, darkened room with eyes open and ears unplugged. Attenuation correction computed tomography (CT) and emission imaging equivalent to that used in the ADNI-3 protocol were performed in a GE Discovery MI PET/CT. FDG images were analyzed by determining volumes-of-interest (VOIs) based on each subject’s individual structural MRI (Hammers N30R83 maximum probability atlas of Neuro Tool of PMOD Biomedical Image Quantification, version 3.9; PMOD Technologies, Ltd., Zurich, Switzerland). Seventy-eight regions were identified. Global metabolism was determined by calculating the volume-weighted average of the standardized uptake values (SUV). To facilitate comparison between subjects, relative regional activity was calculated by normalizing the activity of each region to the subject’s global value and interpreted in a similar manner (e.g., a value of 1.2 = 20% greater activity than the individual’s global mean value).

### 2.5. Statistical Analysis 

One-way ANOVAs were performed to compare volume-weighted average SUVs and to investigate differences in the cognitive and motor task performances between the groups. Post-hoc pairwise analyses (unpaired *t*-tests; Bonferroni correction) and effect sizes (Cohen’s d) were calculated to clarify any significant main effects. The sphericity and normality assumptions were assessed with the Shapiro-Wilk test and Mauchly’s test of sphericity. Greenhouse–Geisser corrections were planned if/when the sphericity assumption was violated. Additionally, due to the exploratory type of this pilot study, unpaired *t*-tests and effect sizes were used to compare the normalized relative regional metabolism between groups [34,49]. Significance was set at *p* ≤ 0.05 and analyses were made using GraphPad Prism 9 (GraphPad Software, San Diego, CA, USA).

## 3. Results

All test variables satisfied the assumptions for the statistical tests and all THC and CBD users completed all of the testing as described above. Two NU subjects were lost-to-contact after the first session and did not complete the imaging session. Thus, the NU PET outcomes have *n* = 14 subjects. Table 1 displays participant demographics. All cannabis users possessed a medical card and used state approved products either as tablets, tinctures, or oils.

### 3.1. Cognitive and Motor Tasks

The ANOVA results indicated a significant main effect of group for the FT-I (F (2,26) = 6.35, *p* = 0.01) and FT-E scores (F (2,26) = 5.59, *p* = 0.01). Post-hoc tests revealed that THC users had poorer scores on the FT-I and FT-E than CBD users (*p* = 0.01, d = 1.65 and *p* = 0.04, d = 1.13, respectively) and NU (*p* = 0.04, d = 1.13 and *p* = 0.02, d = 1.26, respectively). The ANOVA results for all other cognitive and motor tasks revealed no significant differences between groups (F (2,26) ≤ 2.98, *p* ≥ 0.07). Table 2 shows the outcome measures and corresponding statistical results.

### 3.2. Brain Image Analysis

#### 3.2.1. Global FDG SUV

**Figure 1:**
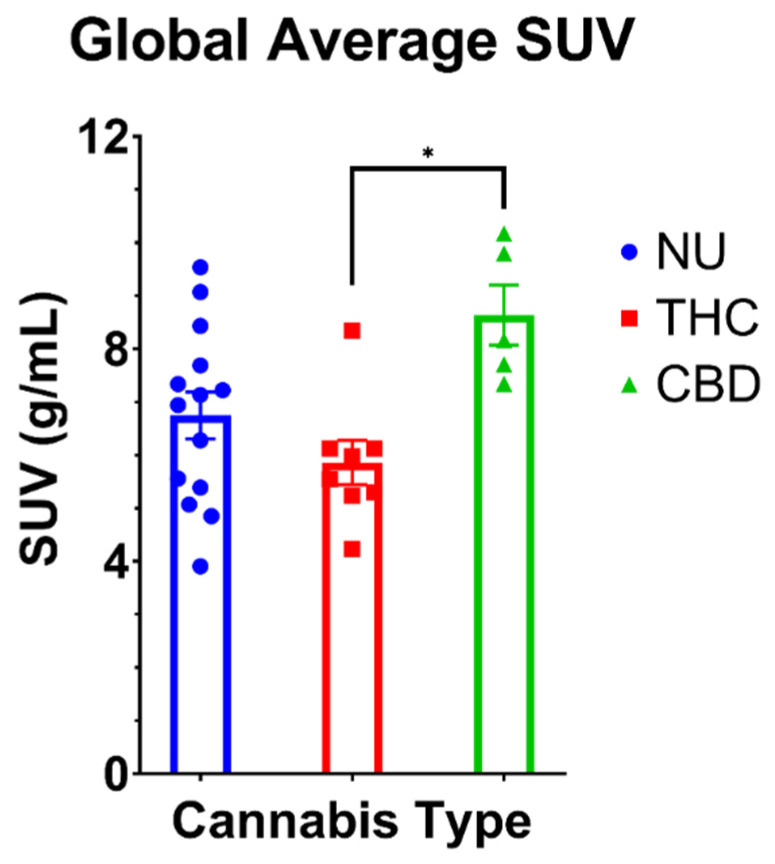
Comparison of volume-weighted global average SUVs for cannabis non-users (NU), THC users and CBD users. Data are mean ± SEM. * CBD users have glucose hypermetabolism (*p* = 0.02, Bonferroni correction) compared to THC users. There were no statistical differences between THC users or CBD users and cannabis non-users.

The ANOVA indicated a significant main effect of cannabis type on volume-weighted average SUVs (F (2,24) = 5.54; *p* = 0.01). Post-hoc analysis revealed higher mean SUV in CBD users compared to THC users (*p* = 0.01; d = 2.29). Comparisons between both cannabis groups and NU revealed no statistically significant differences (THC vs. NU: *p* = 0.54; CBD vs. NU: *p* = 0.06; Figure 1).

#### 3.2.2. Relative Regional Metabolism

**Figure 2:**
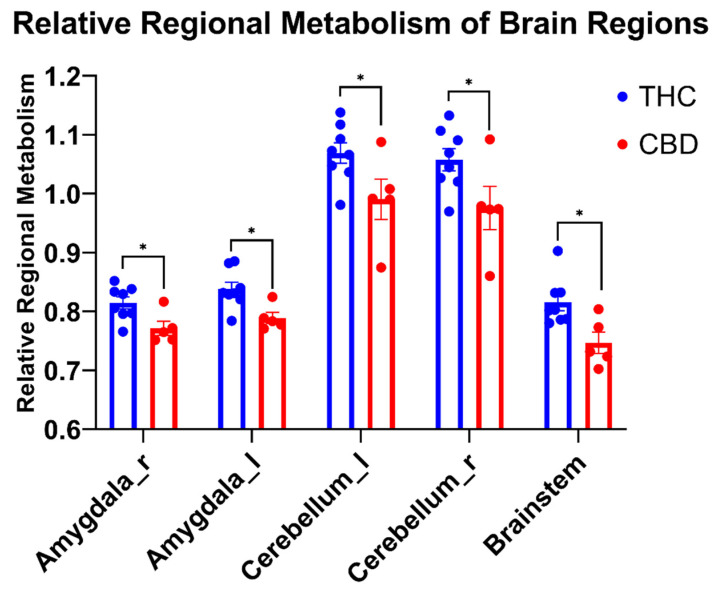
Comparisons of THC and CBD users in relevant brain regions. Data are mean ± SEM. * THC users had significantly higher relative regional metabolism (*p* ≤ 0.05) for all brain regions shown. Amygdala_r, right amygdala; Amygdala_l, left amygdala; Cerebellum_l, left cerebellar lobule; Cerebellum_r, right cerebellar lobule.

Relative regional metabolism was only compared between THC users and CBD users based on the outcomes of the global average SUV ANOVA presented above. Additionally, because performance differences between THC and CBD users were only seen in the FT-I and FT-E, only brain regions relevant to cognition and inhibitory control were compared between cannabis groups. Unpaired *t*-tests (see Figure 2) revealed higher relative metabolism in THC users compared to CBD users in the right and left amygdala (*p* = 0.02, d = 1.59; *p* = 0.01, d = 1.68, respectively), right and left cerebellar lobes (*p* = 0.05, d = 1.26; *p* = 0.04, d = 1.31, respectively), and in the brainstem (*p* = 0.01, d = 1.71).

## 4. Discussion

This is the first study comparing cognitive function, motor function, and cerebral glucose uptake in older (>50 years) chronic THC users, CBD users, and NU. The novel observations were that THC users performed worse than both CBD users and NU on the FT-I and FT-E and these findings were accompanied by lower global FDG SUV and greater relative cerebral glucose uptake in the amygdala, cerebellum, and brainstem of older THC users compared to CBD users. The THC users had lower global metabolism than the CBD users and non-users and a greater proportion of that metabolism was directed toward critical brain regions like the amygdala, cerebellum, and brainstem.

Normal aging is correlated with declines in cognitive abilities, including processing speed, memory, language, and certain visuospatial and executive-functioning skills [31]. A major concern associated with cannabis use in aging populations is that use may increase the risk for cognitive impairment and dementia. Specifically, cannabis use might exacerbate the decline in cognitive processing speed and visual skills that are associated with normal aging, which have distinct consequences for increasing the risk of falls or other injuries [30,31]; yet only a few studies have focused on long-term or chronic effects of cannabis in older adults. A recent review [50] reported two studies with a subject mean age over 65 which investigated cognitive function in cannabis users (current or former) and controls [51,52] and reported no difference in various cognitive measures. When cannabis use was stratified into THC and CBD groups, still no differences were seen in cognitive performance [53]. On the other hand, our study found significant differences only for the Flanker Test (FT-I and FT-E) between older THC users, CBD users, and NU, with THC users performing significantly worse than the other two groups. The Flanker Test is an executive function measure that gauges a subject’s capability to allocate cognitive resources to process external stimuli (attention) and their ability to ignore superfluous stimuli (inhibitory control) [54]. Importantly, some brain regions involved in attention processes and inhibitory control include the amygdala [55] and the cerebellum [56] and the relative activity of these regions was increased in the THC users vs. the CBD users in the current study (Figure 2).

Furthermore, it is known that the main psychoactive component, THC, causes dose-dependent toxicity and structural and functional variations in brain regions rich in cannabinoid receptors (i.e., CB1) such as the cerebellum and amygdala [56]. Moreover, functional and structural changes in the hippocampus/parahippocampus complex and in the amygdala have often been described in chronic cannabis users [57,58,59]. Thus, chronic cannabis use with high THC products is associated with brain morphology alterations in regions linked to memory and executive and affective processing in young adults [58]; the results of the current study suggest this might be the case in older cannabis users as well.

Interestingly, chronic cannabis use has been correlated with affect dysregulation related to amygdala function [60]. For example, several MRI studies have shown structural and functional changes in the amygdala, a key region in emotional processing, after chronic cannabis use. Linked to healthy controls, young adults who used cannabis had lower activation in the amygdala in an emotional arousal word task during functional MRI (fMRI) [61]. Another fMRI study showed that adolescent cannabis users demonstrated greater amygdala activation to angry faces compared to controls [62]. In our study, older THC users had greater activity in the amygdala at rest as indicated by greater relative glucose uptake compared to CBD users. It might be that the Flanker Test induced a greater emotional arousal state (stress) in the older THC users, which might have resulted in poorer performance (Table 2). Chronic cannabis use has also been related to the dysregulation of stress responsivity in humans [63], with studies indicating that chronic use was related to both blunted and hyperactive stress responses [64,65,66]. Cuttler, et al. [66] showed that controls had increased cortisol levels under a stress-provoking condition compared to baseline. They did not notice similar increases in active cannabis users. One more study found that both abstinent and active cannabis users had constant hyperactivity of the hypothalamic-pituitary-adrenal axis (measured by blood cortisol and ACTH levels) compared to healthy controls [64].

The cerebellum comprises more neurons than the rest of the brain [67,68]. Therefore, it is a crucial region when investigating brain function in cannabis users. Specifically, the cerebellum has a high density of cannabinoid receptors (both CB1 and CB2), which are the primary target of THC and its metabolites [67]. Cerebellar involvement in associated processes may be to some extent explained by the vast connections the cerebellum has with other parts of the brain, like the ventral tegmental area [67], striatal zones [69], prefrontal cortices [70], amygdala [71,72] and hippocampus [73,74,75]. These connections make the cerebellum a structure that is likely to be involved in at least some effects of chronic THC administration, including cognitive performance. However, most research on the effects of cannabis and THC on the cerebellum, particularly at the preclinical level, has concentrated on the influence of cannabis and cannabinoids on motor coordination and performance, likely a result of the important and significant role of the cerebellum in motor coordination and planning [76].

Interestingly, cerebellar metabolism of chronic cannabis users in a resting condition was diminished, but activity increased and was associated with the feeling of “being high” after acute cannabis consumption [77]. Furthermore, long-term daily cannabis users showed an increase in cerebellar blood volume [78], which stayed after 4 weeks of abstinence [79]. Because glucose metabolism and regional cerebral blood flow are highly correlated [80], these results are in line with the increased relative glucose metabolism in older THC users found in the present study.

Studies of impaired cognitive function also indicated cerebellar alterations that may be relevant for chronic cannabis use. For example, even though chronic cannabis use did not seem to affect attentional capabilities, cannabis users had altered cerebellar activity while performing an attentional task [81]. The authors also reported that this activity was associated with the estimated total amount of marijuana use and the age of cannabis use onset. Furthermore, a study on short-term memory revealed some insufficiencies in cannabis users that were associated with cerebellar blood flow increases [82]. Another previous study on inhibitory control also described impaired performance in chronic cannabis users and demonstrated heightened associations between the cerebellum and parietal cortices associated with recent cannabis use [83]. Cannabis use was also negatively associated with reward-related decision-making performance in a gambling task, and users displayed enhanced cerebellar activity compared to non-using controls [84]. This form of general cerebellar hyperactivity during decision-making was also reproduced in another study [85].

Along with other brain regions, the amygdala and cerebellum include projections to the brainstem, which mainly controls cardiovascular and respiratory functions. Although CB1 and CB2 receptor density is low in the brainstem [68], altered activity in the brainstem might still affect the outcome of the Flanker Test. Differences in the Flanker Test were only observed between older THC users and CBD users. THC and CBD can have diverse effects on regional brain function [86], which may trigger their different symptomatic and behavioral effects, like CBD’s purported ability to block the psychotogenic effects of THC [85]. Sadaka, et al. [87] found that CBD reduces autonomic arousal under conditions of emotional and physical stress. The authors suggested that CBD induces activation in the prefrontal cortex and deactivation in the cerebellum and brainstem, and that CBD can influence the emotional and cognitive behavior associated with anxious and fearful events. Furthermore, fMRI studies showed that CBD decreases activation in the anterior cingulate, cerebellum, and amygdala in a visual fear paradigm [13].

The current study has several important limitations which must be considered. First, this study has a relatively small sample size; however, because the study was exploratory in nature and pairwise differences were accompanied by large effects sizes, these results can inform future study designs. Still, larger sample sizes are necessary to further elucidate these findings. Additionally, as reported previously [19], the THC content of medical cannabis in Iowa is relatively low compared to other commercial or recreational forms, which limits the generalizability of the results. Moreover, although seven out of eight THC and five out of five CBD subjects reported using cannabis for pain relief, they had various physical impairments that contributed to their pain and various cannabis concentrations and methods of use (e.g., capsules vs. tinctures). This may have resulted in heterogeneous groups, which might also decrease the generalizability of the conclusions. Additionally, the rate of recreational cannabis consumption and poly-drug use (e.g., cannabis in concert with hallucinogens) or concomitant consumption of tobacco and/or alcohol products is high, particularly in chronic pain subjects; thus, the effects of other substances on the results cannot be ruled out. Although biological sex should be considered as a potentially important variable [88], the small sample size prevents any comparisons to determine if sex was relevant to the current results. We also asked subjects to abstain from use of their respective product immediately before the session to avoid acute cannabis effects, but we did not verify their compliance via blood metabolite testing and some acute effects might have influenced the findings. Finally, our study involved subjects who may be more aptly characterized as middle-aged (mean age ~60 years) and therefore the results might not generalize to even older adults.

Future longitudinal studies with larger sample sizes which describe the chronic effects of multiple THC:CBD ratios on functional outcomes and cerebral glucose metabolism are necessary. These studies should be powered to assess early-onset vs. late-onset use (i.e., >30 years vs. 5–15 years) and biological sex. Further research is also needed to investigate the effects of cannabis use on dopamine function in older adults, because of the known effects of cannabis on dopamine function in younger populations [89], and the role of dopamine in general cognition, including reward-based decision making [90]. This potential interaction is also important because age-related changes in the dopaminergic system have been observed [91] and are linked to detrimental mental health and wellbeing outcomes [26,92]. It is also noteworthy that the chemical composition and cannabis constituent profile has changed over time. Specifically, the concentration of THC in recreational cannabis has increased significantly [93,94], thus it is important to establish age of onset of cannabis use when assessing chronic effects.

## 5. Conclusions

Our data suggest that THC and CBD may have unique effect profiles, underscoring the importance of delineating between types of cannabis use in future research. THC content might be responsible, at least in part, for the decreased performance on the Flanker Test observed in the current study. Furthermore, these effects might be accompanied by lower global metabolism and greater relative metabolism in the amygdala, cerebellum, and brainstem of THC users compared to CBD users. Future research utilizing the use of specific diagnostic criteria, pairing of appropriate neurocognitive testing to functional imaging, adequate exposures to cannabis, and defining cannabis type in concert with consumption method may help address the current significant gaps in the literature.

## Figures and Tables

**Table 1:** Subject characteristics for each group. Data are mean ± SD.

	THC Users	CBD Users	Non-Users
*n* (f)	8 (4)	5 (3)	16 (9)
Age (yrs)	59.3 ± 5.7	54.6 ± 2.1	58.2 ± 16.9
Height (cm)	171.1 ± 12.1	171.7 ± 7.5	157.6 ± 41.9
Weight (kg)	89.3 ± 20.5	97.6 ± 24.1	84.2 ± 31.6
Duration of use (yrs)	20.2 ± 8.7	1.4 ± 1.3	n/a
Uses per week (days)	5.6 ± 2.6	5.4 ± 1.5	n/a
Uses per day (times)	1.9 ± 1.1	1 ± 0	n/a
THC:CBD per dose (mg; range)	150:7.5–600:30	14:280–70:1400	n/a

Note: THC = Δ9-Tetrahydrocannabinol, CBD = cannabidiol.

**Table 2:** Summary data for motor outcomes and the results of post-hoc *t*-tests comparing each group.

Study Variables; Mean ± SD				*p*-Value (Cohen’s d)		
	THC Users	CBD Users	NU	THC Users vs. CBD Users	THC Users vs. NU	CBD Users vs. NU
n	8	5	16			
Cognitive tasks						
RT Simple (ms)	355.5 ± 73.1	315 ± 39. 2	328.3 ± 43.4	0.56	0.73	0.99
RT Choice (ms)	642 ± 172.0	507.2 ± 46.4	607.7 ± 79.5	0.12	0.99	0.25
FT-C (ms)	999.5 ± 142.8	852.4 ± 111.3	946.75 ± 108.0	0.12	0.94	0.40
FT-I (ms)	1166.9 ± 171.9	906.8 ± 129.3	1015.6 ± 112.8	0.01 (1.65)	0.04 (1.13)	0.37
FT-E (ms)	167.4 ± 99.1	54.4 ± 48.8	70.5 ± 63.6	0.04 (1.34)	0.02 (1.26)	>0.99
Motor Tasks						
30MWT time (s)	25.4 ± 7.2	26.5 ± 4.1	25.5 ± 5.2	0.99	0.99	0.99
30MWT steps	45.3 ± 8.2	46.8 ± 7.1	47.1 ± 7.6	0.99	0.99	0.99
30MWT velocity (m/s)	1.3 ± 0.3	1.2 ± 0.2	1.2 ± 0.2	0.99	0.99	0.99
AP Pathlength (cm)	2.1 ± 0.3	2.5 ± 1.4	2.4 ± 0.9	0.99	0.99	0.99
ML Pathlength (cm)	0.8 ± 0.2	1.1 ± 0.5	0.9 ± 0.4	0.46	0.99	0.99
COP area (cm^2^)	1.1 ± 0.4	2.5 ± 0.9	8.9 ± 29.1	0.99	0.99	0.99
Pegboard dom (s)	25.2 ± 3.7	22.7 ± 2.7	22.2 ± 3.3	0.60	0.15	0.99
Pegboard nondom (s)	33.2 ± 10.5	36.6 ± 19.9	30.0 ± 12.1	0.99	0.80	0.51
Grip Strength dom (kg)	33.1 ± 9.2	38.1 ± 11.4	31.8 ± 13.2	0.99	0.99	0.95
Grip Strength nondom (kg)	33.2 ± 10.5	36.6 ± 19.9	30.0 ± 12.1	0.99	0.99	0.99
Fall Risk (%)	33.2 ± 45.4	9.8 ± 20.0	5.6 ± 11.9	0.40	0.07	0.99

Note: THC = Δ9-Tetrahydrocannabinol, CBD = cannabidiol, NU = non-users, RT = reaction time, FT-C = Flanker Test compatible, FT-I = Flanker Test incompatible, FT-E = Flanker Effect, 30MWT = 30-m walk test, AP = anterior-posterior, ML = medio-lateral, COP = center of pressure, dom = dominant hand, nondom = nondominant hand.

## Data Availability

The data that support the findings of this study are available on request to the corresponding author.
